# Spontaneous Rupture of Arachnoid Cyst in a Child: A Rare Case Report

**DOI:** 10.7759/cureus.33652

**Published:** 2023-01-11

**Authors:** Abdulelah S Almousa, Sahar N Alotaibi, Muhanad M Al Wadany, Feras M Al Wadany, Ahlam S Alharbi

**Affiliations:** 1 General Practice, King Faisal University, Hofuf, SAU; 2 General Practice, King Saud bin Abdulaziz University for Health Sciences, Riyadh, SAU; 3 Family Medicine, King Faisal University, Hofuf, SAU

**Keywords:** case report, spontaneous rupture, magnetic resonance imaging, subdural hygroma, arachnoid cyst, headache

## Abstract

An arachnoid cyst is a benign lesion filled with cerebrospinal fluid that usually develops in the middle cranial fossa. The arachnoid cyst may become symptomatic if it has a large size or when it gets ruptured. Spontaneous rupture of an arachnoid cyst is a very rare complication. We report the case of an 11-year-old girl who was brought to the emergency department with a complaint of a progressive headache that was associated with vomiting. On examination, she was found to have papilledema. Subsequently, magnetic resonance imaging of the brain was performed to exclude any space-occupying lesion. The scan demonstrated a right extra-axial temporal lesion, measuring 7.8 x 5.4 x 4.9 cm on maximum dimensions, along with an extension to the right cerebral convexity in a crescentic shape. The lesion follows the signal intensity of cerebrospinal fluid on all sequences and exhibited no post-contrast enhancement or restricted diffusion. The lesion exerted a mass effect in the form of compression of the right temporal lobe. These findings were consistent with an arachnoid cyst with subdural hygroma. The patient was referred to the neurosurgery team. Then, the right temporal arachnoid cyst was drained through the right temporal craniotomy and the subdural hygroma was drained through a frontal Burr hole. The patient was seen after one month in the pediatrics clinic and was completely asymptomatic.

## Introduction

An arachnoid cyst is a congenital extra-axial cystic lesion containing cerebrospinal fluid between arachnoid sheets with no communication with the ventricular system. It represents around 1% of all intracranial masses [1]. It can be seen in different locations such as the quadrigeminal and suprasellar cisterns, posterior cranial fossa, and along the cerebral convexity. However, the most common location of an arachnoid cyst is the middle cranial fossa, occurring in over 50% of cases. While the majority of patients with arachnoid cysts are asymptomatic, arachnoid cysts may present with headaches, seizures, and neurological deficits [2]. Arachnoid cysts may get ruptured in the setting of head trauma. Spontaneous rupture of arachnoid cysts is very unusual [3]. Here, we report the case of a young girl with spontaneous rupture of an undiagnosed arachnoid cyst.

## Case presentation

We present a case of an 11-year-old girl who was brought to the emergency department by her parents with a complaint of progressive headache. The headache was in the occipital region bilaterally. It started one month before the presentation and gradually increased in severity. She described the headache as sharp in nature. The headache was non-radiating, and it was worse in the morning. The parents reported that the headache became associated with multiple episodes of vomiting over the past few days. They visited a number of pediatric clinics, and the child was offered simple analgesics with non-steroidal anti-inflammatory drugs. The patient was not known to have any medical conditions. She had no previous surgeries. She was up-to-date on the national vaccination schedule. There was no family history of migraine.

On physical examination, the child appeared to be in pain. There were no signs of respiratory distress. Her vital signs were within the normal range, including a pulse rate of 80 beats/min, a respiratory rate of 18 breaths/min, a blood pressure of 98/54 mmHg, and a normal temperature of 37.0 °C. Neurological evaluation revealed normal findings of the cranial nerves, apart from bilateral papilledema. Examination of the upper and lower extremities showed normal tone, power, reflexes, and coordination. There was no weakness or sensory loss noted on examination.

**Figure 1:**
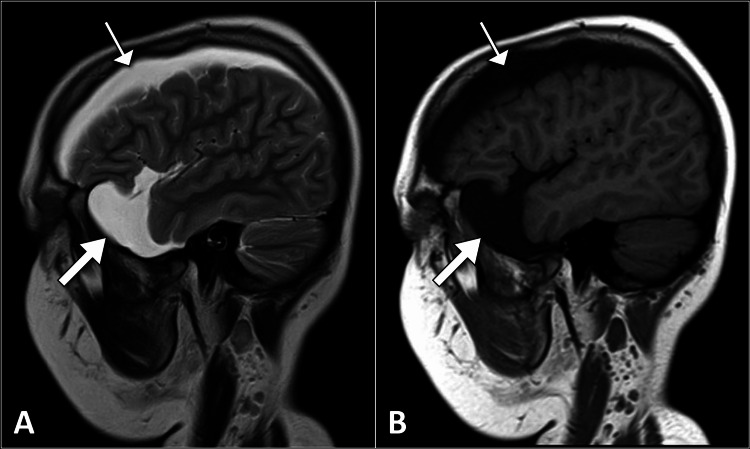
Sagittal brain MRI images show a cystic lesion in the temporal region (thick arrow) with extension along the cerebral convexity (thin arrow), which demonstrates a high signal on the T2-weighted image (A) and a low signal on the T1-weighted image (B) MRI: magnetic resonance imaging

**Figure 2:**
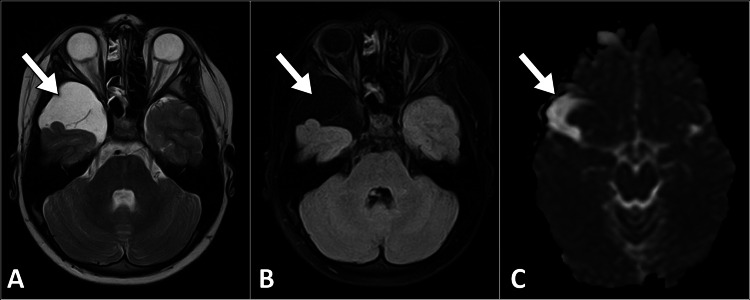
Axial brain MRI images show a lesion in the right middle cranial fossa (arrow) that demonstrates high signal intensity on the T2-weighted image (A) with complete suppression on the FLAIR image (B) and facilitated diffusion on the ADC map (C) These findings are consistent with an arachnoid cyst. MRI: magnetic resonance imaging; FLAIR: fluid-attenuation inversion recovery; ADC: apparent diffusion coefficient

Considering the concerning features of headache, a head computed tomography scan was planned to exclude any space-occupying lesion. Despite discussing the importance of this investigation, the parents strongly refused due to the fear of radiation exposure. Then, the patient underwent brain magnetic resonance imaging. The scan demonstrated a right extra-axial temporal lesion, measuring 7.8 x 5.4 x 4.9 cm on maximum dimensions, along with an extension to the right cerebral convexity in a crescentic shape. The lesion follows the signal intensity of cerebrospinal fluid on all sequences and exhibited no post-contrast enhancement or restricted diffusion. The lesion exerted a mass effect in the form of compression of the right temporal lobe. There was no midline shift or evidence of herniation. No evidence of acute infarction or hemorrhage was noted. The findings were suggestive of an arachnoid cyst in the right middle cranial fossa with a rupture into the subdural space (Figures 1-2).

The diagnosis was discussed with the parents, and the patient was referred to the neurosurgery team for further management. The right temporal arachnoid cyst was drained through the right temporal craniotomy. The subdural hygroma was drained through a frontal Burr hole. The drained cerebrospinal fluid was sent for further analysis. It showed no malignant cells on cytological examination, and there was no growth on the culture. The postoperative course was unremarkable, and the child was discharged on the sixth postoperative day. The patient was seen after one month in the pediatrics clinic and was completely asymptomatic.

## Discussion

We report the case of a spontaneous rupture of a right-sided, middle cranial fossa arachnoid cyst in a young girl. Unlike our case, arachnoid cysts are more common in males and are more likely to occur on the left side. An arachnoid cyst in the middle cranial fossa is usually associated with a mass effect on the temporal lobe. Galassi et al. classified these arachnoid cysts into three types [4]. In type 1, there is a small semicircular cyst in the anterior part of the temporal region. In type 2, there is a medium-sized quadrangular cyst. In type 3, there is a large oval cyst. The clinical manifestations of cysts are closely related to their type since larger cysts are more likely to be symptomatic. The symptoms are related to increased intracranial pressure, neurological deficits, seizure, or even cranial bulges [3].

Arachnoid cyst rupture is rare. Cress et al. conducted a case-control study to identify the risk factors for arachnoid cyst rupture or hemorrhage in children [5]. They identified that cysts larger than 5 cm are more likely to rupture. Also, head injury in the preceding month was a significant factor, even if the trauma was relatively minor. In the present case, the parents gave no history of any previous trauma.

The diagnosis of an arachnoid cyst can be made accurately with cross-sectional imaging like computed tomography or magnetic resonance imaging. The lesion appears as well-circumscribed with similar density and signal intensity to that of cerebrospinal fluid [3]. The surgical management of arachnoid cysts remains controversial with regard to the best approach. Surgical management is indicated for symptomatic cases (e.g., headache). In asymptomatic patients, surgical management should be individualized based on the possible risk of rupture. The reported approaches include craniotomy, cyst shunting, and endoscopic fenestration [1].

## Conclusions

Spontaneous rupture is a very rare complication of an arachnoid cyst. The rupture of the arachnoid cyst leads to the formation of a subdural hygroma and increased intracranial pressure, which mandates surgical intervention. The use of cross-sectional imaging is vital for prompt diagnosis of this potentially life-threatening condition.
